# Resilience of Octocoral Forests to Catastrophic Storms

**DOI:** 10.1038/s41598-020-61238-1

**Published:** 2020-03-09

**Authors:** H. R. Lasker, Á. Martínez-Quintana, L. Bramanti, P. J. Edmunds

**Affiliations:** 1 Department of Environment and Sustainability and Department of Geology, University at Buffalo, Buffalo, NY 14260 USA; 2 CNRS-Sorbonne Université, Laboratoire d’Ecogéochimie des Environnements Benthiques, LECOB, Observatoire Océanologique de Banyuls sur Mer, 1 avenue Pierre Fabre, 66650 Banyuls sur Mer, France; 3 Department of Biology, California State University, 18111 Nordhoff Street, Northridge, CA 91330-8303 USA

**Keywords:** Community ecology, Community ecology, Marine biology

## Abstract

After centuries of human-mediated disturbances, Caribbean reef communities are vastly different from those described in the 1950s. Many are functionally dominated by macroalgae, but this community state represents only one of several possibilities into which present-day coral reefs can transition. Octocorals have always been abundant on Caribbean reefs, but increases in their abundance over the last few decades suggest that arborescent octocorals have the potential to expand their populations on reefs that hitherto had been dominated by scleractinians. Here we show that octocoral-dominated communities at three sites on the fringing reefs of St. John, US Virgin Islands, were resilient to the effects of two Category 5 hurricanes in 2017. We describe the dynamics of octocoral communities over five years at three sites on shallow reefs (~9-m depth), and test for the effects of Hurricanes Irma and Maria. The hurricanes depressed the densities of juvenile and adult octocoral colonies as much as 47%. However, there were only weak effects on species richness and the relative abundances of the octocoral species. The hurricanes did not alter patterns of spatial variability in octocoral community structure that existed among sites prior to the storms. The density of octocoral recruits (individuals ≤ 5 cm high) was reduced in the year following the hurricanes, mainly due to a decline in abundance of recruits <0.5 cm, but returned to pre-storm densities in 2019. Persistently high octocoral recruitment provides a mechanism supporting ecological resilience of these communities. Continuing environmental degradation is a threat to all tropical marine communities, but the reefs of St. John illustrate how “octocoral forests” can persist as the structurally dominant community on Caribbean reefs.

## Introduction

Most present-day coral reefs greatly differ from the reefs described by ecologists in the 1950s and 1960s^1–4^, and are strikingly different from those encountered by European explorers in the 15th Century^5^. These changes have been attributed to a diversity of natural and anthropogenic disturbances^6^, but the emergence of climate change and ocean acidification^7^ has created the possibility that reefs dominated by scleractinian corals might cease to exist within decades^8^. Over the last four decades, Caribbean coral reefs have undergone marked declines in the abundance of scleractinian corals^9–11^. These losses typically have been recorded as stepwise incremental reductions in cover attributed to pulse disturbances caused by hurricanes^9^ and bleaching^12,13^, that overlie the chronic effects of press disturbances such as diseases^14^, depressed rates of growth^15^, and reduced recruitment^16^.

**Figure 1:**
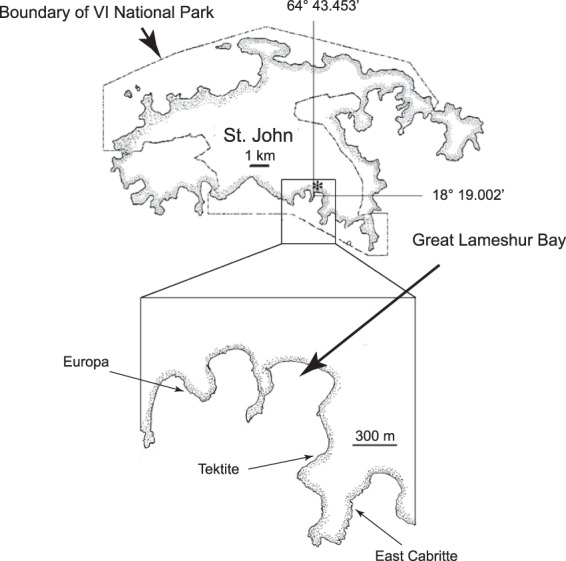
Map showing the location of the study sites on St. John, U.S. Virgin Islands.

The declining abundances of scleractinians have generally been characterized as a trade-off in both the abundance and functional importance of scleractinians and macroalgae^17–19^. However, it has also become apparent that other sessile benthic invertebrates have increased in abundance^20^, and at some sites, where quantitative data are available, octocorals have increased as scleractinians have declined^21–24^. Octocorals have always been abundant on Caribbean reefs^25,26^, but unlike scleractinians, which have undergone marked declines in abundance, octocorals have increased in abundance on shallow reefs (i.e., <30 m depth) at sites ranging from the oceanic, southwest Caribbean^24^, to the U.S. Virgin Islands^22^, and the Florida Keys^23^. The temporal scope of these studies have spanned historic bleaching events and hurricanes, suggesting octocoral communities may be resistant and/or resilient to disturbances that have had strong negative effects on scleractinians. In this study we report on the resistance and resilience of octocorals on three reefs on the south shore of St. John, U.S. Virgin Islands (Fig. 1), following the passage of Hurricanes Irma and Maria in September 2017.

The frequency and severity of coral bleaching events has increased in the last few decades^4,13,27–29^, but prior to the first global scale mass bleaching in 1983^30^, hurricanes were regarded as the major disturbance affecting reefs. The effects of hurricanes on coral reefs have been reported in the scientific literature since the early 20^th^ century^31–33^ and the geologic record reveals that they have been important agents of disturbance on coral reefs for at least 125ky^34^. The presence of tropical reefs from the Pleistocene on which the scleractinian fauna mirrors the taxonomic composition of 20^th^ century reefs^35,36^ is testimony to the historic ability of coral reefs to recover from disturbance. In more recent decades, however, populations of scleractinians on Caribbean reefs have not recovered from major hurricanes^9^. Debate has continued as to whether present day coral reef communities, especially those dominated by macroalgae, represent readily reversed phase transitions or alternative stable states^17,37–40^, and even the generality of the observation and the meaning of “dominance”, have been challenged^18^. However, the extent to which the shift in dominance from scleractinians to other taxa is representative of a stable change has not been discussed. An important determinant of the future of reef communities dominated by taxa other than scleractinians is their stability^38^ and resilience following disturbance.

The shallow reefs on the south shore of St. John, U.S. Virgin Islands, have recently undergone a shift in benthic community structure that favors octocorals^21,22,41–44^. The decline in abundance of scleractinians has been recorded in detail^45–47^. Although octocorals have been less thoroughly studied, octocorals also are known to be negatively affected by hurricanes^22,48^ and bleaching^49^, but the available evidence suggests their populations can recover in only a few years^22^. Tsounis and Edmunds^21^ hypothesized that, unlike scleractinians, the shift in abundances of octocorals has been driven by the resilience of octocorals to major disturbances. In September 2017 Hurricane Irma passed over St. John, U.S. Virgin Islands, as category 5 hurricane followed two weeks later by Hurricane Maria, also a category 5 storm which passed south of St. John; together, these storms are unprecedented within human memory for the region. The waves, heavy rainfall, and resultant terrestrial runoff associated with these storms provided an opportunity to assess the effects of the hurricanes on the emerging octocoral-dominated communities we have been studying for six years. If present-day octocoral-dominated communities in the Caribbean are resistant and resilient to disturbance, then the trend towards community dominance by octocorals may create a “new normal” for shallow reefs in the region: a persistent transition from scleractinian reefs to octocoral forests (*sensu* Rossi *et al*.^50^).

## Results

**Figure 2:**
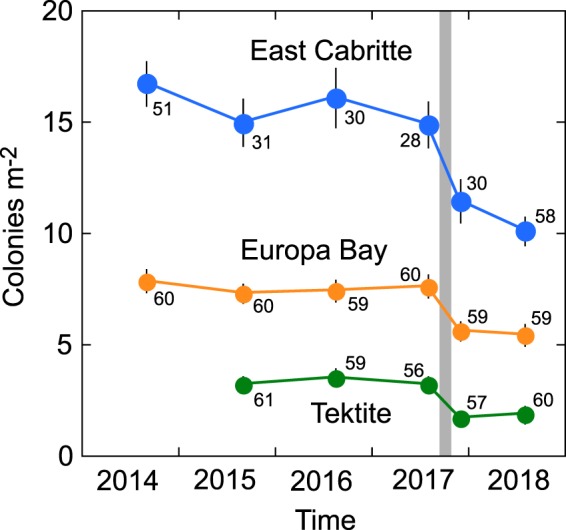
Densities of juvenile and adult octocoral colonies (pooled among taxa) at three sites on the south shore of St. John from 2014 to 2018. Sampling began in July 2014 at East Cabritte and Europa Bay, and in July 2015 at Tektite, it continued annually to July 2018, and included November 2017 after Hurricanes Irma and Maria (shaded bar). Means ± SE shown (unless error bars are smaller than the symbols) with number of 1 m^2^ quadrats that were censused shown next to each symbol.

Starting in 2014, we conducted annual surveys of octocoral abundance at three sites on the south shore of St. John, U.S. Virgin Islands. Those surveys have continued through 2019 and include an additional survey in November 2017, two months after Hurricanes Irma and Maria. Surveys differentiated between recruits (1 polyp to colonies 5 cm tall), and all colonies >5 cm tall that included juveniles (i.e., colonies that are not sexually mature), and adult colonies (i.e., colonies that are sexually mature). In 2014, the abundances and richness of juvenile and adult colonies differed among sites, with 30 taxa and 15.9 ± 0.5 colonies m^−2^ in East Cabritte, 31 taxa and 7.6 ± 0.2 colonies m^−2^ in Europa Bay, and 20 taxa and 3.4 ± 0.2 colonies m^−2^ in Tektite (mean ± SE). The mean densities at any one site differed 1–11% between any pair of years between 2014 and July 2017, and did not significantly differ among times at any site prior to the hurricanes (Fig. 2, Supplementary Information Table 1). Hurricanes Irma and Maria struck St. John in September 2017, causing reductions of 23–47% in mean octocoral densities (pooled among taxa) of juvenile and adult colonies at all sites, with the largest effect at Tektite. Abundances in November 2017 and July 2018 were the lowest observed at each site and comparisons of estimated marginal means for each site and census show that abundances in the November 2017 and/or 2018 were lower than all other censuses at each site (Supplementary Information Table 1). Post-hoc comparisons of abundances in each year showed that the November 2017 densities were lower than densities recorded in all other censuses (Supplementary Information Table 1). The decline in abundance that was associated with the hurricanes affected colonies across the entire range of colony heights. Size frequency distributions of octocorals differed among sites. However, the size frequency distributions did not significantly differ before and after the hurricanes (Supplementary Information Fig. 1), indicating that effects on structural complexity created by different size colonies and biomass (which is related to colony size) paralleled the changes in colony abundances.

**Figure 3:**
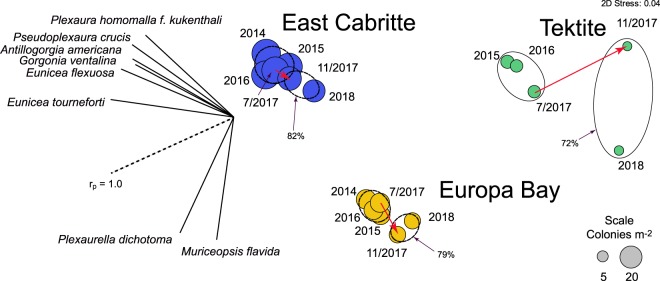
Two-dimensional ordination by non-metric multidimensional scaling (nMDS) showing relationships among sampling dates based on octocoral abundances by taxon, site, and time. Densities (colonies m^−2^) were square-root transformed and used to prepare resemblance matrices using Bray-Curtis dissimilarities. Significance among times was evaluated using SIMPROF with significant clusters (P ≤ 0.05) shown as similarity contours (East Cabritte 83%, Europa Bay 79% and Tektite 72%). Red arrows shows transition associated with Hurricanes Irma and Maria. Vectors show the association (as Pearson correlations) of the abundance of the eight most common taxa with the two nMDS axes.

Multivariate analysis of community structure based on juvenile and adult colonies identified large differences among sites and much smaller differences associated with the hurricanes (Fig. 3). Prior to the hurricanes, the octocoral communities at each site were 77–89% similar in sequential years (based on Bray-Curtis dissimilarities), while similarities among pairs of the three sites varied from 42% (in November 2017, Tektite vs East Cabritte) to 68% (Europa Bay vs East Cabritte in August 2018). The hurricanes had small but significant effects on the community structure of each site (SIMPROF, P-perm ≤ 0.05). However, differences among sites remained consistent following the hurricanes, and Bray-Curtis dissimilarities of the communities before and after the hurricanes indicate they were 75 to 84% similar. Octocoral species richness in the two post-hurricane surveys (November 2017 and August 2018) was similar to that recorded in all surveys before the hurricanes (East Cabritte, 36 vs 33 taxa; Europa Bay, 34 vs 31; and Tektite, 25 vs 24). Species that were not found after the hurricanes had been uncommon and had not regularly been recorded in the earlier surveys (Supplementary Information Table 2).

**Figure 4:**
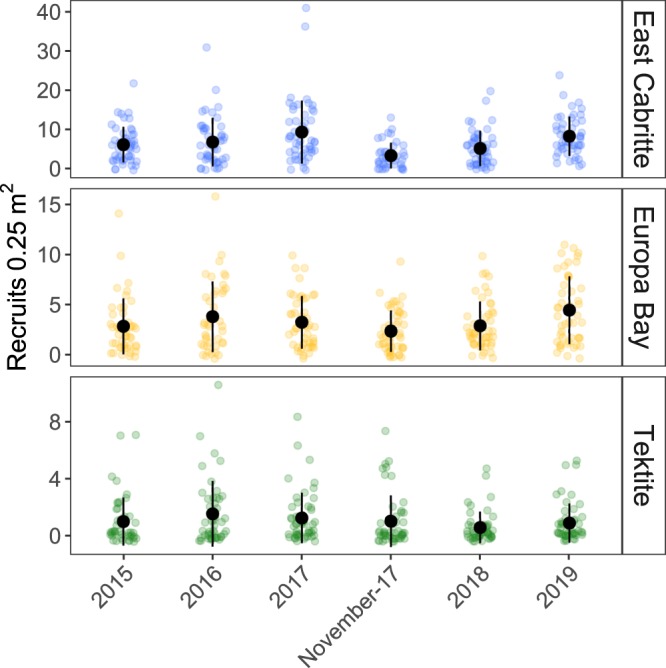
Density of octocoral recruits found at each of 3 sites in July, from 2015 through 2019. The 2018 survey was conducted 10 months after the passage of Hurricanes Irma and Maria. Color dots represent number of recruits found in each 0.25 m^2^ quadrats. Darker points indicate multiple quadrats with the same recruit density. Black dots and bars represent mean and standard deviations for each site and year. Note that each site has a different scale.

Densities of octocoral recruits at the three sites on St. John were quite variable during the 3 years prior to Hurricanes Irma and Maria, ranging from 6.1 (±0.9 SE) to 9.32 (±1.3) recruits 0.25 m^−2^ at East Cabritte, 2.8 (±0.4) to 3.3 (±0.5) at Europa and 1.0 (±0.1) to 1.6 (±0.2) at Tektite (Fig. 4). Numbers of recruits found in any single quadrat ranged from 0 to 41 with a median of 2 recruits and a mode of 0. The hurricanes had the greatest effect on recruit densities at East Cabritte. Recruit densities at East Cabritte were lower in November 2017 than in all other censuses, (Fig. 4, Supplementary Information Tables 3 and 4). Recruitment in 2018, the following summer, was still lower than 2017 prior to the hurricanes, but not significantly different from 2015 or 2016. Recruitment in 2019 was greater than in 2018 but not significantly greater than in 2017. Examination of recruit size frequency distributions (Supplementary Information Fig. 2) indicate that most of the decline and recovery in recruitment following the hurricanes can be attributed to changes in number of ≤1 cm recruits. Recruit densities at Europa followed a somewhat similar pattern to that observed at East Cabritte. The lowest number of recruits were observed in November 2017 (2.4 ± 0.3) and the greatest in 2019 (4.5 ± 0.5). However, both the recruit densities and the magnitude of the differences in recruitment between years were lower than at East Cabritte. The only significant pairwise differences among the Europa recruit censuses were the comparisons of 2019 with 2015, 2018 and November 2017 (Supplementary Information Table 4). Recruitment at Tektite was the lowest of the three sites and there was no significant difference among the censuses (Supplementary Information Table 4).

## Discussion

Understanding the stability of octocoral communities relative to disturbance is a critical component in assessing whether octocoral “animal forests” are the stable successor to the scleractinian dominated communities that preceded them. Although monitoring that also follows octocoral abundances is relatively rare, increased abundances of octocorals have now been documented on shallow reefs in the Caribbean such as the Florida Keys^23^, Virgin Islands^21,22^ and the southwest Caribbean^24^. Disturbance events and whether octocoral communities are affected (i.e. resistance) and/or whether they can rebound from the disturbance (i.e., resilience^51^) provide opportunities to assess the longer term stability of the octocoral forests. The octocoral communities that we studied on St. John suffered dramatic declines in abundances in association with the passage of Hurricanes Irma and Maria, and in that sense, were not resistant to the effects of severe storms. However, they showed resistance in species composition as the effects on the relative abundances of octocoral species were small (Fig. 3), and the differences between species composition before and after Hurricanes Irma and Maria were much smaller than the differences between the three sites.

Octocoral recruitment following the hurricanes facilitates the resilience of the octocoral community. The number of recruits found in the November 2017 census at East Cabritte was 82% of the number found in 2017 and 64% of the average of the preceding 3 years. However, much of that decline was among the smallest (≤1 cm) recruits (Supplementary Information Fig. 2) and that drop may reflect the high mortality that naturally occurs among recruits^52,53^ that would have settled in the summer. The more meaningful comparison is between pre-hurricane recruit abundances with the numbers of recruits observed in 2018 and 2019. In July 2018, recruit densities were lower than in 2016 and 2017 by 21% and 47%, respectively. That change was driven by a decline in the density of the smallest size class recruits (<0.5 cm) which declined by 77% in 2018 (Supplementary Information Fig. 2). In 2019, densities of recruits <0.5 cm recovered to levels similar to the years previous to the hurricanes with 1.82 ± 0.37 (mean ± SE) recruits 0.25 m^−2^.

The drop in abundance of the smallest size class following the hurricanes could have occurred through a variety of mechanisms. There may have been a reduced supply of larvae, perhaps due to lower fecundity following the hurricanes. Such effects are known from bleaching in scleractinians^54,55^. Whether stress from hurricanes might cause similar effects is not known, but the allocation of resources to repair colony damage should affect resources available for reproduction^56^. Alternatively, the hurricanes may have altered the substratum making it less suitable for successful settlement. Grazing and browsing affect recruit survival^52,53^ and changes in the abundance of predators or in the ratio of predator abundance and prey could have depressed the abundance of recruits following the hurricanes. Indirect effects such as the reduction of predators of the taxa that feed on octocorals^57^ could also account for the lack of the smallest recruits in 2018. Any explanation must also account for the differences in responses at the three reefs. Regardless of the causation of the reduced numbers of recruits in 2018, the return to pre-hurricane recruitment will, barring other disturbances, regenerate the populations over time. The rapid recovery of recruitment rates following the hurricanes plays a critical role in supporting the resilience of the octocoral community. Reduced adult densities might also stimulate growth of all colonies through a reduction in resource competition^58^ and reduced interference created by abrasion of colonies against each other^59^.

Under the environmental and biotic conditions prevailing on present-day shallow Caribbean reefs, octocoral communities have exhibited resilience and some resistance^51,60^ to major disturbances including hurricanes (present data), bleaching^61^, eutrophication^24^, diseases^62^ and early signs of the effects of ocean acidification on seawater pH^63,64^. At high population densities, Caribbean octocorals form underwater forests^50^ that alter environmental conditions, such as light, currents, and sedimentation, beneath their canopy, and create habitat utilized by many species^50,65,66^. Octocoral forests, unlike scleractinians, do not build hard, wave resistant structures, but they provide some of the ecosystem services formerly supported by scleractinians (e.g., providing fish habitat^67^, acting as a carbon sink^68^ and generating sediments^69^). In the present environmental conditions, octocoral-dominated communities may constitute the “new normal” for shallow Caribbean reefs. However, it is important to recognize that the resilience that these communities currently exhibit may decline with future intensification of hurricanes, bleaching events, and other disturbances, making the current communities a temporary state in a longer term sequence that has been described as a “slope to slime”^8^.

## Materials and methods

Juvenile and adult surveys of octocorals in St. John have been previously described^42^. The three sites, East Cabritte (18° 18.360′N, 64° 43.140′W), Europa Bay (18° 19.016′N, 64° 43.798′W) and Tektite (18° 18.796′N, 64 43.356′W) are located on the southern shore of St. John, within the Virgin Islands National Park. All three sites are fringing reefs. Quantitative assessments of the environments at East Cabritte and Europa have previously been discussed^42^. The substratum at East Cabritte is a mix of igneous boulders and scleractinian-generated carbonate mounds. The reefs at Europa and Tektite are all carbonate with a mix of carbonate rock (living and mostly dead coral heads) and sand patches. East Cabritte is more exposed to wave action, which dominantly comes out of the east and southeast, than Europa^42^. Those differences affect light levels and sedimentation with East Cabritte having lower rates of sedimentation than Europa. Tektite is the most protected of the three sites, but there was extensive evidence of mobilization of loose rubble at the site during the hurricanes.

Censuses of juvenile and adult colonies were conducted annually in the summers of 2014 through 2018. Those data were supplemented with additional surveys in November 2017, two months after Hurricanes Irma and Maria. At each site, abundances of juveniles and adult octocorals were monitored along 6 permanently marked 10-m long transects, spaced at 10 m intervals. Transects were oriented perpendicular to the shore, and ranged in depth from 5.6 to 9 m. During each juvenile and adult census, 1 m^2^ positions along the transects were randomly chosen, each on a randomly selected side of the transect. Ten quadrats were censused on each transect at Europa and Tektite. At East Cabritte, where octocoral densities were high, 6 quadrats per transect were sampled during the 2015–2017 censuses. Rarefaction analysis indicates the sample sizes were suitable for assessing species richness^42^. The identity and height of all arborescent octocoral colonies that were ≥5-cm tall were recorded. *Erythropodium caribeorum* and encrusting *Briareum asbestinum* were not included in the surveys. *Eunicea laxispica, E. mammosa* and *E. succinea* could not be unambiguously distinguished in the field and those counts were pooled into a single operational taxon, as were *Pseudoplexaura flexuosa* and *P. wagenaari*. Recruit censuses were conducted in July from 2015 to 2019. Recruits (1 polyp – 5 cm tall) were enumerated, and when possible were identified to genus in separate 0.25 m^2^ quadrats randomly placed (n = 8) along each transect at each site (48 quadrats per site). Multivariate analyses were conducted with Primer version 6^70^. Using quadrats as replicates, recruit density and adult/juvenile density data were analyzed using the Generalized Linear Methods function in SPSS (v 26).

## Supplementary information

Supplementary Information.

## Data Availability

Data used in the study are available through the Biological and Chemical Oceanography Data Management Office (BCO-DMO), https://www.bco-dmo.org/dataset/682966/data; 10.1575/1912/bco-dmo.751176.1; and 10.1575/1912/bco-dmo.765328.1.
