# Supplementary material for: Resilience of Octocoral Forests to Catastrophic Storms
Source: Sci Rep. 2020 Mar 9;10:4286. doi: 10.1038/s41598-020-61238-1 (PMC7063042; doi:10.1038/s41598-020-61238-1)
Supplement: Supplementary file 1 — Supplementary Information. [file 41598_2020_61238_MOESM1_ESM.pdf]

Resilience of Octocoral Forests to Catastrophic Storms

Lasker[1]* HR, Martínez-Quintana[1] Á, Bramanti[2] L, Edmunds[3] PJ

Supplementary Information

Table 1. ANOVA* of octocoral densities (ln transformed) in 1 m^2 quadrats from censuses July 2014 through July 2018 at 3 sites on St. John Virgin Is.

Source	Type III Sum of Squares	df	Mean Square	F	Sig.
Site	184.938	2	92.469	359.009	<0.001
Year	14.991	5	2.998	11.641	<0.001
Site * Year	2.620	9	.291	1.130	0.338
Error	214.812	834	.258		
Total	3533.491	851			

Homogeneous Groups of Years

	Year	N	Group 1	Group 2	Group 3
Student-Newman-Keuls	November 2017	146	1.6076		
	2018	150		1.8581	
	2015	152		1.8771	
	July 2017	144		1.8958	
	2016	148		1.9088	
	2014	111			2.3933

Homogeneous Groups of Sites

	Site	N	Group 1	Group 2	Group 3
Student-Newman-Keuls	Tektite	267	1.2628		
	Europa	356		1.9494	
	East Cabritte	228			2.5821

Estimated marginal means - Site by Year

Site		Mean Ln(Density)	Std. Error	95% Confidence Interval	
				Lower Bound	Upper Bound
East Cabritte	2018	2.299	0.067	2.168	2.429
	November 2017	2.427	0.093	2.246	2.609
	2015	2.682	0.091	2.504	2.861
	2017	2.697	0.096	2.509	2.886
	2016	2.736	0.093	2.554	2.918
	2014	2.781	0.071	2.641	2.920
Europa	2018	1.753	0.067	1.622	1.884
	November 2017	1.766	0.066	1.636	1.896
	2016	2.027	0.066	1.897	2.157
	2015	2.036	0.066	1.907	2.164
	2017	2.043	0.066	1.914	2.171
	2014	2.064	0.066	1.935	2.193
Tektite	November 2017	1.012	0.067	0.880	1.144
	2018	1.285	0.087	1.114	1.456
	2015	1.312	0.065	1.184	1.439
	2017	1.338	0.068	1.205	1.471
	2016	1.370	0.066	1.240	1.500

* ANOVA – Univariate analysis of variance function in SPSS v 26

Table 2. Octocoral species present in monitoring surveys

	East Cabritte						Europa						Tektite				
	2014	2015	2016	2017	Nov 2017	2018	2014	2015	2016	2017	Nov 2017	2018	2015	2016	2017	Nov 2017	2018
Antillogorgia americana	X	X	X	X	X	X	X	X	X	X	X	X	X	X	X	X	X
Antillogorgia acerosa		X	X	X	X	X		X	X	X	X	X	X	X	X	X	X
Antillogorgia hummelincki										X							
Antillogorgia rigida	X	X	X		X	X	X	X	X	X		X	X	X	X	X	X
Briareum asbestinum (upright form)	X	X		X		X	X	X	X	X	X	X	X	X	X	X	X
Eunicea asperula		X	X	X	X	X	X				X	X				X	
Eunicea calyculata	X	X	X	X	X	X	X	X	X	X	X	X	X	X	X		
Eunicea calyculata f. coronata	X					X											
Eunicea clavigera	X	X	X	X	X	X	X	X	X	X		X	X				X
Eunicea flexuosa	X	X	X	X	X	X	X	X	X	X	X	X	X	X	X	X	X
Eunicea fusca	X	X	X	X	X	X	X	X	X	X	X	X		X	X		
Eunicea knighti					X												
Eunicea lacinata	X	X	X	X	X	X	X	X	X	X	X				X		

Eunicea laxispica/mammosa/succinea	X	X	X	X	X	X	X	X	X	X	X	X	X	X	X	X	X
Eunicea palmeri	X	X													X		
Eunicea pinta		X															
Eunicea pallida				X													
Eunicea succinea f. plantaginea	X	X	X				X						X	X	X		
Eunicea tayrona	X	X	X	X	X	X	X	X	X	X	X	X	X	X	X	X	
Eunicea tourneforti	X	X	X	X	X	X	X	X	X	X	X	X					X
Eunicea tourneforti f. atra	X	X	X	X	X	X	X	X		X	X						X
Gorgonia mariae		X					X	X					X				X
Gorgonia ventalina	X	X	X	X	X	X	X	X	X	X	X	X	X	X	X	X	X
Muricea atlantica	X		X	X	X	X	X	X	X	X	X	X					
Muricea elongata								X		X	X	X	X	X			
Muriceopsis flavida		X	X	X	X	X	X	X	X	X	X	X	X		X		
Muricea muricata	X	X	X		X	X	X	X	X	X	X	X					
Muricea pinnata															X		
Pseudoplexaura crucis	X	X	X	X	X	X	X	X		X	X	X					X
Pterogorgia citrina	X	X	X	X		X	X	X	X	X	X	X					
Plexaurella dichotoma	X	X	X	X	X	X	X	X	X	X	X	X	X	X	X	X	X

Pseudoplexaura flagellosa/wagenaari	X	X	X	X	X	X	X	X	X	X	X	X	X	X	X	X	X
Plexaurella grisea	X	X		X		X	X		X	X		X	X		X		X
Pterogorgia guadalupensis	X	X	X			X	X	X	X	X	X	X					
Plexaura homomalla	X	X	X	X	X	X	X	X	X	X	X			X	X	X	
Plexaura kukenthali	X	X	X	X	X	X	X	X	X	X		X	X	X	X	X	X
Plexaura kuna	X	X	X	X	X	X	X	X	X		X	X	X	X	X	X	X
Plexaurella sp.	X				X		X	X	X					X	X	X	
Plexaurella nutans	X	X	X	X		X	X	X	X	X	X	X	X	X	X	X	X
Pseudoplexaura porosa	X	X	X	X	X	X	X		X	X	X	X		X	X	X	X
Antillogorgia sp.																	
Eunicea spp.	X	X	X	X	X	X	X	X	X	X	X	X	X	X	X		X
Muricea spp.	X						X	X									
Pterogorgia spp.								X									
Pseudoplexaura spp.	X		X	X				X	X			X		X			

Table 3. Generalized Linear Analysis* of recruit densities in 0.25 m^2 quadrats at three sites on St. John, U.S. Virgin Islands.

Tests of Model Effects

Source	Wald Chi-Square	df	Sig.
(Intercept)	17599.768	1	0.000
Site	411.954	2	0.000
Census	42.445	5	0.000
Site * Census	30.323	10	0.001

Estimated Marginal Means 1: Site

			95% Wald Confidence Interval	
Site	Mean*	Std. Error	Lower	Upper
East Cabritte	1.2298	.01359	1.2032	1.2565
Europa	1.0605	.01356	1.0339	1.0870
Tektite	.8391	.01371	.8122	.8659

Estimated Marginal Means 2: Census

			95% Wald Confidence Interval	
Census	Mean*	Std. Error	Lower	Upper
2015	1.0274	.01899	.9901	1.0646
2016	1.0651	.01956	1.0267	1.1034
2017	1.0921	.01872	1.0554	1.1288
2018	1.0155	.01992	.9765	1.0546
2019	1.1049	.01885	1.0680	1.1418

Nov 2017	.9537	.01950	.9155	.9920

Estimated Marginal Means 3: Site* Census

Site	Census	Mean*	Std. Error	95% Wald Confidence Interval	
				Lower	Upper
East Cabritte	2015	1.2325	.03198	1.1698	1.2952
	2016	1.2290	.03335	1.1637	1.2944
	2017	1.3472	.03198	1.2845	1.4099
	2018	1.1779	.03335	1.1126	1.2433
	2019	1.3467	.03264	1.2827	1.4107
	Nov 2017	1.0457	.03622	.9747	1.1167
Europa	2015	1.0257	.03335	.9604	1.0911
	2016	1.0836	.03490	1.0152	1.1520
	2017	1.0644	.03264	1.0004	1.1284
	2018	1.0555	.03335	.9901	1.1208
	2019	1.1424	.03264	1.0784	1.2063
	Nov 2017	.9913	.03231	.9280	1.0546
Tektite	2015	.8239	.03335	.7585	.8892
	2016	.8826	.03335	.8173	.9480
	2017	.8648	.03264	.8009	.9288
	2018	.8132	.03669	.7413	.8851
	2019	.8256	.03264	.7616	.8896
	Nov 2017	.8242	.03264	.7603	.8882

*Analysis based on natural log of the square root of density using a normal distribution in the GLM function in SPSS v. 26.

Table 4. Kruskal-Wallis comparison of numbers of recruits found in 0.25 m^2 quadrats (48 per site and time) in censuses between 2015 and 2019 at three sites on the south shore of St. John, U.S. Virgin Islands. November 2017 census conducted 2 months after the passage of Hurricanes Irma and Maria. Pairwise values are probabilities that the 2 time periods had the same recruitment. Significant values ($p<0.05$) are in bold. Probabilities are not adjusted for multiple comparisons. Bonferroni correction sets the critical value for rejection of the hypothesis at p= 0.003. Censuses are listed in order of decreasing recruit abundance. Letters in parentheses identify homogeneous groups identified in stepwise Kruskal-Wallis comparisons.

EAST CABRITTE (Kruskal-Wallis test of independence across all censuses, p< 0.001)

	2017	2016	2015	2018	November 2017
2019 (A)	0.930	0.058	**0.039**	**0.004**	**<0.001**
2017 (A,B)		0.067	**0.046**	**0.004**	**<0.001**
2016 (A,B,C)			0.904	0.316	**0.003**
2015 (B,C)				0.367	**0.004**
2018 (B,C)					**0.044**
November 2017 (D)					

EUROPA (Kruskal-Wallis test of independence across all censuses, p= 0.043)

	2016	2017	2018	2015	November 2017
2019 (A)	0.247	0.129	**0.015**	**0.015**	**0.002**
2016 (A,B)		0.729	0.543	0.228	0.063
2017 (A,B)			0.784	0.372	0.118
2018 (A,B)				0.540	0.204
2015 (A,B)					0.516
November 2017 (B)					

TEKTITE (Kruskal-Wallis test of independence across all censuses, p=0.532)

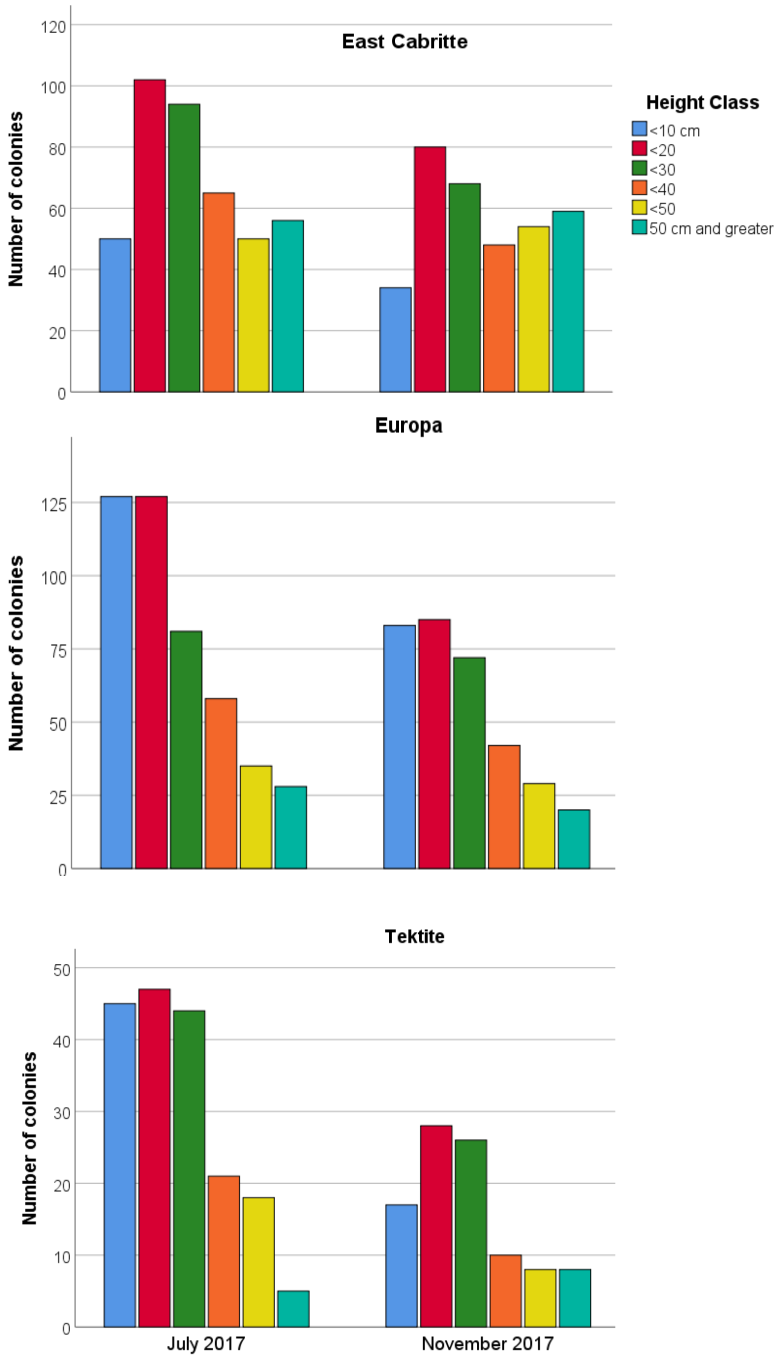

Figure 1. Size-frequency distributions of octocoral colony heights at three sites on St. John before (July 2017) and after (November 2017) Hurricanes Irma and Maria. Distributions differ among sites (Log-linear analysis, site × height, partial Chi square = 110.4, df = 10, $p < 0.001$) but not by year (Loglinear analysis, height × year, partial Chi square =7.1, df = 5, p = 0.22; Likelihood ratio analysis for each site - East Cabritte, Likelihood ratio = 5.51, df = 5, p = 0.36; Europa Bay, Likelihood ratio = 2.73, df = 5, p = 0.74; Tektite, Likelihood ratio = 9.04, df = 5, p = 0.30)

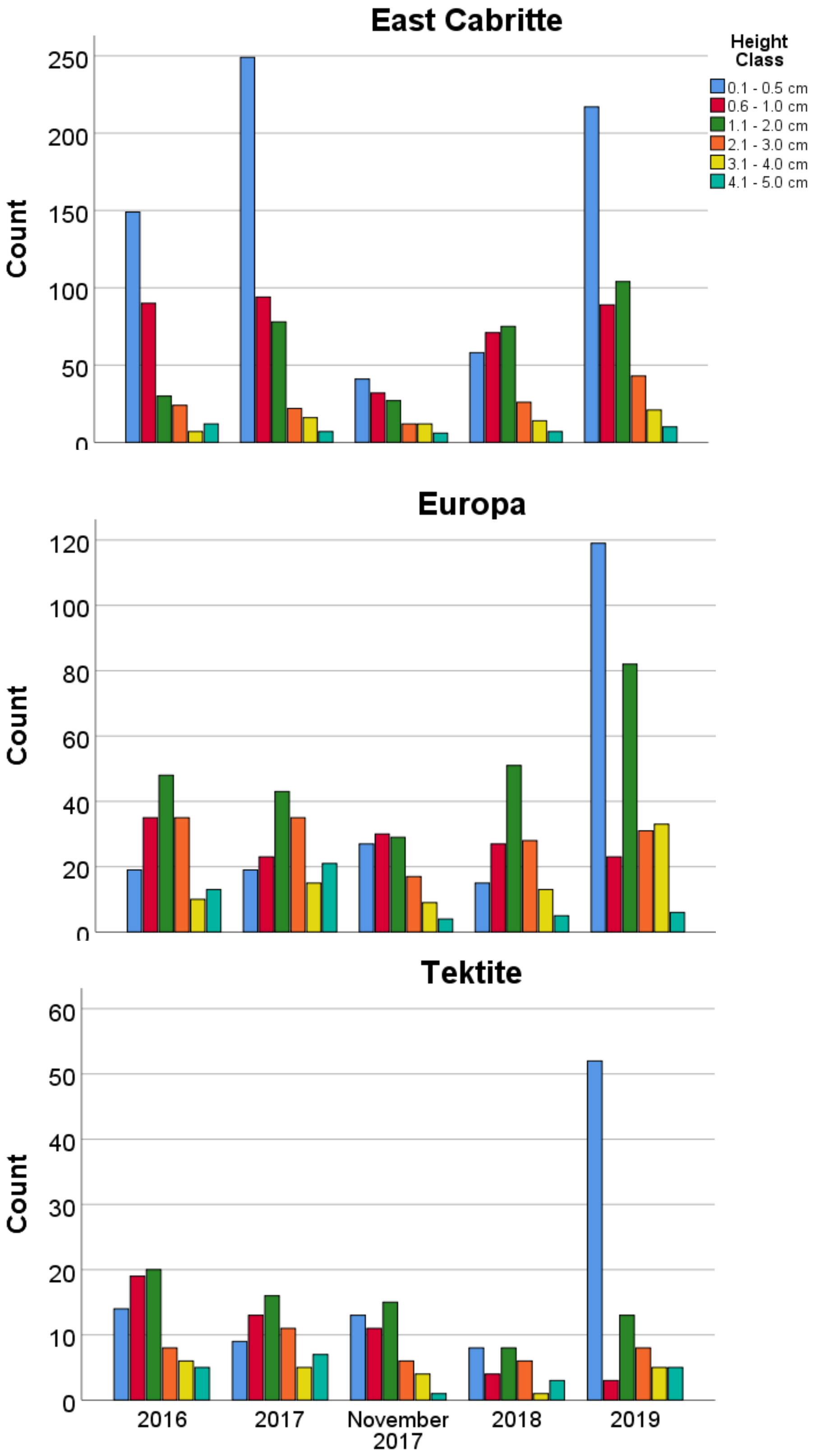

Figure 2. Size frequency distributions of recruits at 3 sites on St. John, U.S. Virgin Is.
